# Microglial Activation in Nociplastic Pain: From Preclinical Models to PET Neuroimaging and Implications for Targeted Therapeutic Strategies

**DOI:** 10.3390/ijms262411861

**Published:** 2025-12-09

**Authors:** Flaminia Coluzzi, Lynda Zeboudj, Maria Sole Scerpa, Anna Giorgio, Roberto Alberto De Blasi, Marzia Malcangio, Monica Rocco

**Affiliations:** 1 Department of Surgical and Medical Sciences and Translational Medicine, Sapienza University of Rome, 00189 Rome, Italy; roberto.deblasi@uniroma1.it (R.A.D.B.); monica.rocco@uniroma1.it (M.R.); 2 Unit Anesthesia, Intensive Care and Pain Therapy, Sant’Andrea University Hospital, 00189 Rome, Italy; mscerpa@ospedalesantandrea.it; 3 Wolfson Sensory, Pain and Regeneration Centre, King’s College London, Guy’s Campus, London Bridge, London SE1 1UL, UK; lynda.zeboudj@kcl.ac.uk (L.Z.); marzia.malcangio@kcl.ac.uk (M.M.)

**Keywords:** microglia, nociplastic pain, neuroinflammation, PET imaging, fibromyalgia, palmitoylethanolamide

## Abstract

Nociplastic pain has recently been introduced as a third entity for identifying patients suffering from chronic pain that arises from altered nociception, without evidence of peripheral nociceptors activation or alterations of the somatosensory system. Currently, the main challenge of nociplastic pain is that it remains a diagnosis of exclusion, since no specific biomarkers are available. Positron emission tomography (PET) neuroimaging studies, using selective translocator protein (TSPO) radiopharmaceuticals, specific for microglia activation, showed a strong correlation between neuroinflammation and nociplastic pain: in particular, in fibromyalgia (FM), which is the prototype disease. Neuroimaging studies identified key brain changes associated with pain processing and neuroinflammation in patients suffering from widespread pain, often associated with sleep, mood disorders, and cognitive impairment. The present review will provide an overview on the role of neuroinflammation in nociplastic pain, focusing on preclinical evidence of microglia activation and advances in PET neuroimaging. Understanding the role of neuroinflammation could have relevant implications in selecting targeted therapeutic strategies and improving analgesic efficacy.

## 1. Introduction

Nociplastic pain (NcplP) is defined as “pain that arises from altered nociception despite no clear evidence of actual or threatened tissue damage that causes peripheral nociceptors activation or evidence of disease or lesion of the somatosensory system causing the pain” by the International Association for the Study of Pain (IASP). Such a term was used in 2016 for the first time, differentiating it from nociceptive and neuropathic pain [1]. NcplP appears in several painful conditions, such as fibromyalgia (FM); neurological disorders, namely multiple sclerosis (MS) [2], Parkinson’s disease (PD) [3], cerebral palsy [4] and especially migraines [5]; orofacial pain [6,7] and complex regional pain syndrome (CRPS) [8]. Visceral pain syndromes may also be associated with NcplP: in particular, irritable bowel syndrome (IBS) [9], pelvic pain, endometriosis [10] and interstitial cystitis (IC)/painful bladder syndrome [11]. Whether pain in cancer [12,13], osteoarthritis [14], rheumatoid arthritis [15,16,17], or lower back pain (LBP) [18] may have a nociplastic nature is still under debate, with conflicting data. Therefore, the overall prevalence and incidence of NcplP is not easy to estimate, as epidemiological data are available for the different diseases rather than for the type of pain.

FM is the most frequently reported health condition associated with NcplP, potentially identifiable as its “prototype”. FM is believed to have a prevalence of 2–4% in the entire population, especially at the working age, with a 9:1 ratio between female and male patients [19], increasing with age [20]. However, first symptoms may appear during childhood [21]. Patients with FM suffer from widespread pain, as well as other signs and symptoms, including headaches, muscle and joint stiffness, increased sensitivity to touch or temperature, mood issues like depression and anxiety and digestive problems, with great impact on patients’ functionality and quality of life (QoL) [22]. FM-derived healthcare costs are high and vastly correlated with FM diagnosis taking years to be achieved, during which patients undergo reiterated—and most times, unnecessary—medical investigations [23,24]. Currently, the diagnosis is often delayed more than 3 years from the moment symptoms firstly arise [25]. Besides clinical examination not being decisive [26], as for many other conditions associated with NcplP [27], the lack of specific biomarkers and laboratory tests make FM difficult to diagnose and can lead to under evaluation [28]. NcplP and FM are now diagnoses of exclusion [29], and their management still remains a challenge in everyday clinical practice [30].

Numerous preclinical and clinical investigations established the role of neuron-glia-immune interactions in pain chronification [31,32]. Neuroinflammation has been shown to play a key role in the pathophysiology of FM. Central nervous system (CNS) neuroplasticity is thought to be the common pathway for different chronic overlapping pain conditions observed in FM patients. Indeed, neuroinflammation and central sensitization are the main pathophysiological mechanisms for developing most NcplP syndromes and different neurodegenerative disorders [33]. Neuroinflammation is characterized by the activation of glial cells and production of inflammatory mediators that potentiate neurotransmission. Preclinical studies have demonstrated that the inhibition of microglial activation alleviates FM symptoms [34].

**Figure 1:**
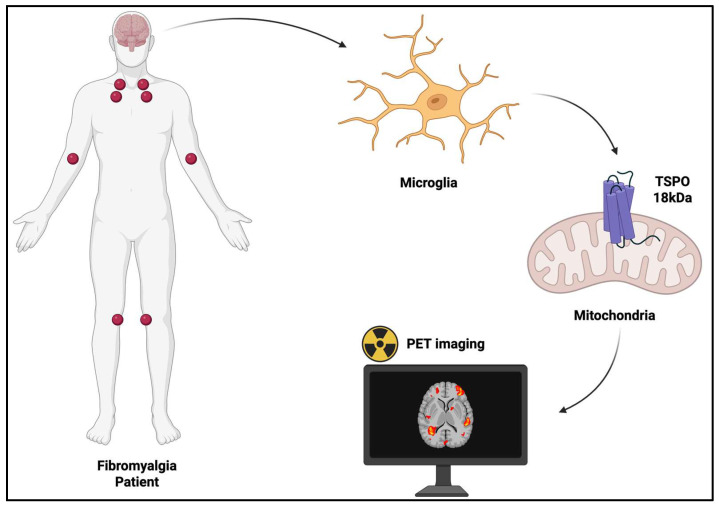
Role of PET imaging for targeting neuroinflammation in nociplastic pain (created using BioRender.com).

In the last few years, pathophysiological mechanisms of NcplP have been investigated using imaging for research purposes; in particular, positron emission tomography (PET) has been applied for detecting neuroinflammation as a potential biomarker of NcplP (Figure 1).

The aim of our review is to highlight the role of microglia activation in the pathogenesis of NcplP, as proven by PET neuroimaging investigation.

## 2. Methods

An extensive literature search was performed in databases such as the PubMed, Google Scholar, Scopus, EMBASE, and Cochrane databases, focusing on the most recent available articles published in the last twenty years. The search strategy used a combination of different keywords, including “microglia”, “neuroinflammation”, “nociplastic pain”, “fibromyalgia” and “PET neuroimaging”.

The research was carried out by two authors independently. The same search strings were adapted for each database and Boolean operators (AND, OR) were applied. The reference lists were manually searched to further investigate and evaluate additional relevant articles.

## 3. Nociplastic Pain and Neuroinflammation

The exact nature and the causes of NcplP are still under debate [35]. Several mechanisms underlying NcplP have been proposed. Peripheral mechanisms include upregulation of sodium channels and sympatho-afferent coupling [36], while spinal mechanisms involve the convergence of signals from different pain loci, regional clustering and amplified spinal reflex transmission, as well as decreased spinal inhibition, winding up, temporal summation and spinal cord reorganization [37,38,39]. Supraspinal mechanisms include hyperresponsiveness to pain stimuli and hyperactivity of brain regions known to be responsible for pain perception: namely, the anterior cingulate cortex (ACC), the medial rostral prefrontal cortex (mPFC), secondary somatosensory cortices and the thalamus. Accordingly, decreased activity and connectivity within the brain circuits responsible for pain inhibition, such as the connection between ACC, mPFC and insula, may be present. Moreover, an increase in glutamine and substance P levels, alongside an inhibition in GABAergic transmission, may occur [36,40]. According to recent findings, such phenomena may also be sex-related and specific, at least in animal models [41,42]. NcplP has been linked to emotional stress, especially related to psychological trauma at a young age [41], with onset signs and symptoms being poor sleep quality, panic disorders and social phobia, rather than merely pain [43]. Accordingly, an association between NcplP and mood disorders has been observed in adults, supporting the biopsychosocial model of pain.

No definitive diagnosis is possible, since no confirmatory test has been established yet for NcplP nor FM [44]. Over time, several interleukins, pro-inflammatory and neurosensitizing mediators, such as IL-6 and IL-8 [45], IL-1β, histamine, TNF-α, calcitonin gene-related peptide (CGRP) and substance P have been implicated in NcplP; these are released by activated thalamic mast cells, thus stimulating thalamic nociceptive neurons directly or through microglia activation in the diencephalon [46,47]. Neurotrophines, such as nerve growth factor (NGF) [48,49] and brain-derived neurotrophic factor (BDNF) [50] have been investigated and measured, both in the cerebrospinal fluid (CSF) and plasma in patients with FM; however, the results were contradictory [51,52]. In FM conditions, CSF cytokine profile features increase in IL-8 and CX3CL1, which appear specific to FM since, for instance, in rheumatoid arthritis CSF, it is the IL-1β level that increases [53]. Since the most likely source of CSF cytokines is microglia, these observations support the possibility that these cells are activated in FM conditions.

Neuroinflammation is recognized as a possible common link between pain and sickness behavior in NcplP [54]. Several triggers, including traumatic events, infections [55,56] and environmental exposure to toxins may promote neuroinflammation processes in NcplP [36]. Microglial activation and neuroinflammation, for instance, have been implicated as putative causes of chronic pain [57,58] and persistent depressive and cognitive symptoms [59] in patients after COVID-19. The growing body of literature, indeed, claims an inflammatory condition [60] and dysregulation of the immune system as underlying mechanisms for chronic pain [61], including NcplP [62,63]. Neuroinflammation has been implicated as being a possible intriguing target of treatment for different chronic pain syndromes [63,64,65].

The recent literature also demonstrates that genetic factors are possibly responsible for up to 50% of FM susceptibility, associated with genes that are implicated in neuronal development, pain processing, and modulation pathways [66]. Moreover, epigenetic alterations may trigger neuroinflammatory responses and central sensitization [67], which is a key factor in the genesis of chronic pain syndromes [68].

## 4. Microglia Activation in Preclinical Models of Nociplastic Pain

### 4.1. Microglial Activation—Shift Pro/Anti-Inflammatory

Microglia, CNS resident macrophages, can sense changes in nearby microenvironment and rapidly become activated. In models of nociceptive and neuropathic pain following peripheral tissue or nerve damage, clusters of microglia located in the dorsal horn of the spinal cord respond to excitation of the first sensory synapse between primary afferent central terminals and dorsal horn neurons. Thus, sensory neuron sensitization (peripheral sensitization) results in enhanced input from the periphery and an increase in synaptic activity in the dorsal horn (central sensitization). Peripheral and central sensitization correlate with behavioral responses to painful stimuli, such as hyperalgesia (increased pain in response to painful stimuli) and allodynia (pain in response to nonpainful stimuli). Spinal cord microglia contribute to central sensitization by clustering at the first sensory synapse and undergoing transcriptional and morphological activation. Pharmacological or genetic inhibition of microglia attenuates hyperalgesia and allodynia in animal models of nociceptive and neuropathic pain by reducing p38 phosphorylation and cytokine production [69]. However, microglial activation, which can be sex-dependent [70,71], is not a uniform process but rather dynamic and heterogenous, evolving with time after injury. Thus, microglia can facilitate neuropathic pain development immediately after injury and remission several weeks after injury [72,73].

Indeed, microglia respond to (i) extracellular ATP via activation of P2Y12, P2X4 and P2X7 receptors; (ii) dorsal horn neuron-derived chemokines, such as CX3CL1, which activates microglial CX3CR1 receptor; and (iii) sensory neuron-derived CSF-1 and Galectin-3 that activate CSF1R and TLR4 receptors in microglia, respectively [61]. Following activation, microglia change morphology and establish direct communication with neurons by the release of factors such as BDNF, TNF-α and cathepsin S (CatS) that in turn activate neurons, establishing a positive feedback loop that maintains nociceptive signaling. Thus, dorsal horn microglia acquire a pronociceptive (pro-inflammatory) phenotype and interventions to deplete microglia or interact with microglia targets and exert anti-nociceptive effects. However, during maintenance and recovery phases of pain, a distinct subset of microglia acquires a reparative and antinociceptive phenotype function and therefore supports the resolution of chronic pain. Indeed, a CD11c+ microglial population in the spinal cord promotes pain resolution through clearance of myelin debris and the release of anti-inflammatory mediators such as IGF-1 [73]. This temporal shift from pronociceptive to an antinociceptive phenotype highlights the dual role of microglia in both pain persistence and remission, suggesting that targeted modulation of microglial functions, rather than global inhibition, may offer a more effective therapeutic approach.

### 4.2. Preclinical Evidence of Neuroinflammation in Nociplastic Pain

In NcplP conditions, widespread pain and altered nociception (hyperalgesia and allodynia) are not associated with tissue or nerve damage. Therefore, in NcplP, peripheral and central sensitization can occur, though they cannot be explained by nociceptive or neuropathic pain mechanisms [74]. For instance, in models of NcplP, allodynia and hyperalgesia are associated with peripheral sensitization of nociceptive neurons and reduced skin innervation. However, dorsal horn microglia are not necessarily activated, unlike in nociceptive and neuropathic pain conditions.

#### 4.2.1. Fibromyalgia

Specifically, in a mouse model of FM after transfer of IgGs from people with FM, nociceptive hypersensitivity is not associated with microglia activation in the dorsal horn of the spinal cord. Instead, in another model, the hypersensitivity that follows the transplantation of gut microbiota from women with FM is accompanied by the activation of microglia.

Indeed, FM IgG intraperitoneal injection in male and female mice induces hypersensitivity to mechanical and cold stimuli within 24–48 h after injection that lasts for more than one week. FM IgGs sensitize unmyelinated C fibers (nociceptors) in the skin and exert a local effect in the dorsal root ganglia, where they bind to and increase the activity of satellite cells, but not neuronal cell bodies. FM IgGs remain in the periphery, as they do not enter the brain and spinal cord, where microglia do not show any sign of response to peripheral fiber sensitization. Such a microglia-independent peripheral pain state resembles a model of neuropathic pain after nucleus pulposus apposition to the sciatic nerve that induces hypersensitivity to mechanical, cold and hot stimuli, but no activation of spinal microglia [75].

Differently, in the repeated cold stress (RCS) model of FM, spinal cord microglia may play a functional role, considering that microglia proliferation and activation can be detected in the spinal cord, starting with the dorsal horn from day 5 after RCS, and pharmacological inhibition of microglia attenuates RCS pain-like behavior [76].

Convincing evidence for microglia activation can be found in a model of FM after transplantation of fecal microbiota from women with FM into germ-free mice. However, since germ-free mice display global defects in microglia, microglial changes should be interpreted with caution [77]. Fecal microbiota transplantation results in skin and muscle, but not visceral, pain hypersensitivity that develops at 4 weeks from transplantation and persists for several weeks. At the same time point, spinal cord microglia are activated and contribute to pain hypersensitivity in this model. Such evidence of microglia activation in an animal model of FM resembles the neuroinflammation that is detected in the brains of people with FM and widespread musculoskeletal pain.

#### 4.2.2. Irritable Bowel Syndrome

**Figure 2:**
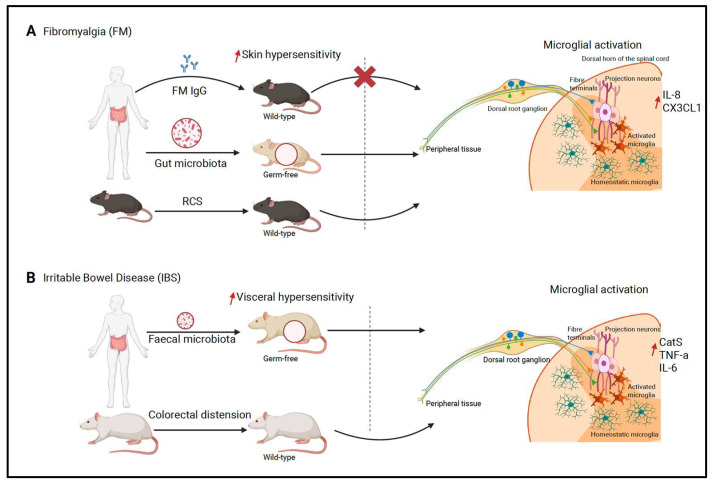
Dorsal horn microglia activation in rodent models of nociplastic pain. (**A**) Microglial activation state during FM animal models, using human IgG transfer into wild-type mice, transplantation of fecal microbiota into germ-free mice and in the repeated cold stress model. (**B**) Transplantation of fecal microbiota from IBS patients into germ-free mice produces visceral hypersensitivity and spinal microglial activation, associated with elevated CatS, TNF-α and IL-6 expression. (**A**,**B**) Spinal cord homeostatic microglia are activated by increased activity of the synapse between sensory neuron fiber terminals and projection neurons. Sensory neuron cell bodies are in the dorsal root ganglia and project axons to peripheral tissue (skin and viscera) and the dorsal horn of the spinal cord (created using BioRender.com).

Another example of NcplP is IBS, where primary visceral pain constitutes a major issue. IBS may onset after gastrointestinal infections and/or antibiotic treatments and regional and diffuse pain hypersensitivity is attributed to central sensitization and alteration in pain processing in the CNS, such as diminished descending modulation [78]. Microglia play a key role in abnormal gut–brain interaction, as suggested by the following observations: the gut microbiota affects microglia and, in models of IBS visceral hypersensitivity, the brain and dorsal horn microglia increase in number and are activated [79,80,81,82,83]. Transplantation of fecal microbiota from patients with IBS into GF rats can lead to visceral hypersensitivity and intestinal inflammation. Moreover, spinal cord microglia are activated, showing morphological changes [84]. In another rat model of IBS following colon-rectal distension at day 8 and 14 after birth, followed by visceral hypersensitivity at 8–10 weeks, microglia are activated in the dorsal horn of the T13-L2 and L6-S2 spinal cord. For example, microglia upregulate and release the protease CatS, which liberates the chemokine domain of neuronal CX3CL1 that activates the CX3CR1 receptor and downstream p38 MAPK in microglia [85]. CatS inhibitors attenuate visceral hypersensitivity, suggesting that the CatS/CX3CL1/CX3CR1 pathway plays a mechanistic role in the development of pain in a model of IBS. Consistently, with the relevance of such a microglia–neuron communication pathway in pain mechanisms, CatS inhibitors and CX3CR1 receptor antagonists attenuate neuropathic pain [86] (Figure 2).

Functional brain imaging in IBS has helped gathering information about pathophysiology, treatment efficacy and biologically based patient subgroups [87]. Gut inflammation can lead to low-grade neuroinflammation in the brain [88]. Gut microbiota dysregulation in IBS can increase intestinal permeability and the release of inflammatory mediators that can reach the brain and activate microglia. However, research on how gut bacteria and microglia communicate to produce pain is still in its early stages, although microbial products such as LPS, short chain fatty acid and tryptophan metabolites can modulate microglial activation. Gut microglia communication likely occurs indirectly through peripheral immune or vagal pathways and establishing causal mechanisms; linking gut dysbiosis to central inflammation remains an important challenge for future research [89].

Together, these findings position microglia as potential effectors of the microbiota–gut–brain axis in NcplP disorders, underscoring the need for mechanistic studies that integrate microbiome profiling, neuro-immune phenotyping and longitudinal symptom tracking in patients.

## 5. Microglia Activation in PET Neuroimaging

PET represents a powerful and non-invasive tool for the detection of brain changes in patients affected by neurological diseases and for the evaluation of the efficacy of treatments [90,91,92].

By using selective radioligands, PET enables precise mapping of molecular and cellular changes, including altered metabolism, receptor availability and dysregulation of the neurotransmitter system across different brain regions. Beyond its diagnostic capabilities, PET imaging can be used as a potential biomarker for clinical outcomes, such as pain intensity, cognitive and affective symptoms, QoL and so on. It also facilitates the assessment of individual responses to therapeutic trials, supporting the development of personalized treatment strategies. Integration of the PET findings with clinical and behavioral data provides a comprehensive understanding of the mechanisms underlying FM-related dysfunction. Furthermore, PET’s sensitivity to changes in neuroinflammatory activity enables the monitoring of treatment effects over time, offering a valuable tool for both mechanistic studies and the evaluation of novel therapies.

As reported in 2025 by Abernavoli and coworkers [93], PET imaging can be used to study brain functions in FM patients using different type of tracers, starting from the most common [^18^F]FDG and also dopaminergic system tracers ([^11^C]Raclopride, [^18^F]Fallypride or [^18^F]DOPA), tracers of GABA_A_ receptors ([^18^F]Flumazenil), ligand of μ-opioid receptor ([^11^C]Carfentanil) and TSPO-targeting tracers ([^11^C]PK11195, [^11^C]PBR28 or [^18^F]DPA-714).

**Figure 3:**
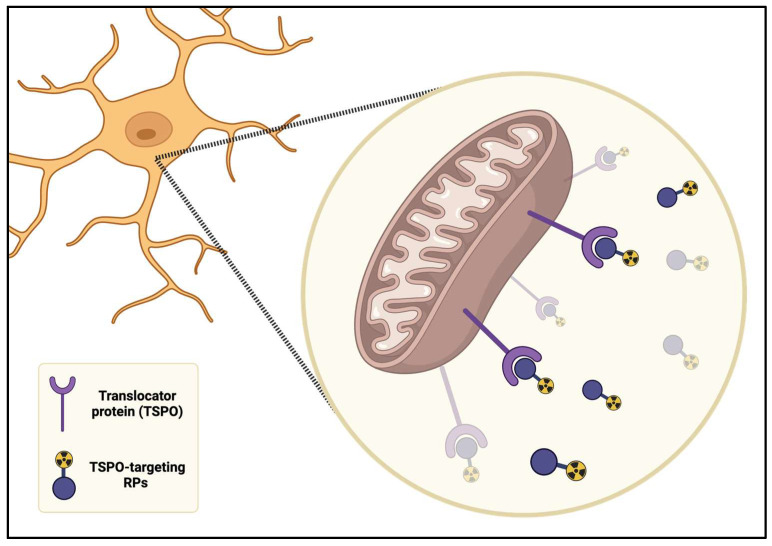
TSPO-targeting RPs on mitochondria of active microglia as a biomarker of neuroinflammation in nociplastic pain (created using BioRender.com).

The TSPO-targeting radiopharmaceuticals (RPs) have been used to evaluate microglia activation, as a marker of neuroinflammation in NcplP (Figure 3).

### 5.1. TSPO-Targeting Radiopharmaceuticals in FM

TSPO is an 18 kDa translocator protein that is expressed in activated microglia and astrocytes and localized on the mitochondrial membrane in steroidogenic cells. In the brain, TSPO’s expression results upregulated in the neuroinflammation conditions, in response to glial cell activation. Due to this, TSPO is extensively studied as a biomarker of neuroinflammation caused by different pathologies, such as FM, chronic pain, neuropathies and neurological diseases including Alzheimer’s disease (AD), PD and MS. Consequently, many radiotracers that are useful in PET imaging have been developed so far.

The development of RPs for PET imaging of the TSPO represents a great challenge. First, these RPs need to cross the blood–brain barrier (BBB): it is facilitated by low molecular weight (lower than 500 Da), lack of formal charge, low hydrogen-binding capability and reduced lipophilicity [94]. Reduced lipophilicity helps a PET RP to cross the BBB for three main reasons. Firstly, very lipophilic molecules tend to bind strongly to plasma proteins and to brain tissue non-specifically, which lowers the free fraction that can reach the target and creates a high background signal [95]. Secondly, high lipophilicity often makes a compound a better substrate for efflux transporters; therefore, the brain-to-blood clearance is accelerated, and the overall brain uptake drops [95]. Third, moderate lipophilicity strikes a balance that allows for sufficient membrane permeability while keeping nonspecific binding low. Many successful PET RPs were designed with only modest lipophilicity to achieve adequate BBB penetration without the penalty of excessive non-specific binding [96,97].

Most of the RPs actually reported are radiolabeled with radionuclides like ^11^C or ^18^F for PET applications [98]. It is possible to describe different TSPO-targeting RPs as useful for neuroinflammation evaluation, including the first- (like [^11^C]PK11195) and second-generation radioligands (like [^11^C]PBR28 and [^18^F]DPA-714) (Table 1). The second generation TSPO RPs have been developed to overcome problems of the so-called first generation ones, like high lipophilicity, which results in highly non-specific in vivo binding [99].

Currently, three PET studies are available in the literature, which evaluated, through the activity of TSPO, if patients with NcplP exhibit higher levels of neuroinflammation than healthy controls (HCs) [100,101,102].

Firstly, in 2019, Albrecht and coworkers investigated neuroinflammation in FM patients, from two different institutes, using [^11^C]PBR28, a TSPO-selective radioligand [100]. The cohort comprised 31 FM patients and 27 HCs, scanned using [^11^C]PBR28, along with a subset (11 FM patients and 11 HCs) that was examined using another radioligand, [^11^C]-L-deprenyl-D2, a tracer predominantly reflecting astrocytic activity, but not microglial signal. Results revealed widespread cortical elevations in TSPO binding in FM patients relative to HCs. The most prominent increases were localized to the medial and lateral walls of the frontal and parietal lobes. Crucially, [^11^C]-L-deprenyl-D2 PET signal revealed no significant group differences, even in regions with elevated [^11^C]PBR28 signal in patients. These data suggested that microglial activation, rather than astrocytic activation, drives the observed neuroinflammatory signatures in FM patients. Overall, this investigation provides the first in vivo evidence of microglial activation in the brains of FM patients. These insights support the potential of glial modulation as a therapeutic target in FM, warranting further studies to confirm astrocytic involvement and explore intervention strategies.

Secondly, in 2021, Seo and coworkers conducted a comparative PET study using [^11^C]-(R)-PK11195 in 12 FM patients compared with 11 patients affected by CRPS and 15 HCs [101]. They found higher neuroinflammation levels in the brains of FM patients compared to CRPS patients. In more detail, FM patients exhibited increased neuroinflammation in the precentral and postcentral gyrus compared to both CRPS patients and HCs. These regions are implicated in abnormal pain sensitivity and impaired modulation within ascending and descending pain pathways, suggesting a potential link between neuroinflammation and FM neuropathology. Conversely, lower neuroinflammation was observed in the medulla, left amygdala and left superior temporal gyrus of FM patients. Previous reports of reduced cortical thickness and gray matter (GM) volume in these and related regions may reflect neuronal loss or dysregulation of inhibitory pain mechanisms, potentially contributing to decreased neuroinflammation [101]. Reduced medullary neuroinflammation may also be associated with altered descending pain control. Correlation analyses indicated that higher affective pain levels were linked to increased neuroinflammation in the left medial frontal cortex, left superior parietal cortex and left amygdala [103,104,105]. Additionally, elevated stress and post-traumatic stress disorder levels were associated with greater neuroinflammation [106,107], suggesting that psychological factors may drive pathological changes in FM [108]. These findings support the notion that FM and CRPS involve partly different neuropathological mechanisms, with abnormal neuroinflammation serving as a key factor; however, the number of patients included was relatively too low to draw conclusions. Moreover, characterizing region-specific changes may be useful for understanding the pathogenesis and development of targeted treatments for both FM and CRPS.

Finally, in 2023, Mueller and coworkers investigated neuroinflammation in FM patients using [^18^F]DPA-714, a second-generation radioligand targeting TSPO [102]. Compared to the 20 min half-life of ^11^C-labeled compounds, the 110 min half-life of ^18^F RPs has several advantages: it allows for delayed imaging, simplifies imaging logistics and permits batch production of the tracers. In this study, 15 women with FM and 10 age-matched HCs underwent PET neuroimaging. The aim was to evaluate whether individuals with FM exhibit neuroinflammation as measured with [^18^F]DPA-714. Increased neuroinflammation was detected in the bilateral precuneus, bilateral postcentral gyri, bilateral parietal and occipital GM, bilateral supramarginal gyri, right temporal GM and the left isthmus of the cingulate gyrus in FM patients compared with HCs [102]. Neuroinflammation may contribute to FM symptoms through central sensitization, where increased neural excitability and reduced inhibition heighten pain responses. Activated microglia release cytokines (IL-1β, IL-6 and TNF-α) and reactive oxygen species that amplify pain signaling and damage neurons, potentially affecting regions like the parietal cortex involved in sensory and cognitive processing. In this study, greater [^18^F]DPA-714 uptake in the right parietal GM was linked to more severe pain, cognitive impairment and lower QoL. A reduction in [^18^F]DPA-714 uptake within the left cingulate gyrus emerged as an unexpected finding, given its involvement in emotional aspects of pain processing and pain perception.

As reported by Kosek et al. in 2016 [109], FM patients who possess a single nucleotide polymorphism in the TSPO gene (rs6971) that confers a high affinity for [^18^F]DPA-714 binding to the TSPO protein report more severe symptoms and higher pain intensity in response to nociceptive stimulation, with respect to other FM patients. The same group of patients exhibit reduced pain inhibition, as reported by Fanton and coworkers [110], supporting the view that genetic polymorphisms related to glial activation may influence FM symptomatology.

To conclude, all three radioligands reported above present almost the same results: the increased TSPO binding in FM patients in the precentral and postcentral gyri, supramarginal gyrus and parietal lobes. However, the use of [^18^F]DPA-714 revealed several additional regions compared to other radioligands and offers the advantage of enabling such imaging in facilities without an on-site cyclotron for ^11^C production.

Given that TSPO upregulation is a common feature of numerous neuroinflammatory disorders, including MS, AD and related conditions, the current generation of TSPO radioligands is unlikely to provide diagnostic specificity for FM and other forms of NcplP. Therefore, rather than having a diagnostic role, these tracers may serve a confirmatory role in demonstrating CNS involvement and ongoing neuroinflammation in FM patients. TSPO-targeted PET imaging, indeed, retains considerable value as a research modality for elucidating the neuroimmune mechanisms underlying FM.

### 5.2. TSPO-Targeting Radiopharmaceuticals in Bowel Disease

TSPO was initially investigated as a marker of microglial activation in neuroinflammation; however, increasing evidence indicates that it also plays a key role in peripheral inflammatory processes, including those affecting the gastrointestinal tract. Although preclinical studies suggested microglia activation as a mechanism underlying IBS, TSPO-targeting RPs have only been used in inflammatory bowel disease. PET imaging investigations consistently demonstrated TSPO overexpression in inflamed mucosa, particularly in epithelial cells, macrophages and infiltrating neutrophils, where it is associated with enhanced oxidative stress, cytokine release and cell death pathways [111,112,113,114,115].

Preclinical PET imaging with TSPO radioligands has revealed the ability to detect and quantify intestinal inflammation more specifically than conventional metabolic tracers such as [^18^F]FDG. Meanwhile, genetic and pharmacological studies highlight that TSPO modulation can influence both disease severity and tissue repair. These findings support the concept that TSPO is not simply a passive marker but an active player in mucosal inflammation and they provide a strong rationale for extending TSPO-based imaging approaches to functional gastrointestinal disorders such as IBS, where low-grade immune activation and altered gut–brain signaling are thought to contribute to pathophysiology. By enabling non-invasive assessment of immune activity in the gut, TSPO PET could offer a novel tool to better characterize IBS subtypes and to monitor therapeutic interventions [116].

## 6. Implications for Therapeutic Strategies

Given the role of activated microglia in neuroinflammation, as shown by PET neuroimaging, it could represent a novel potential target for managing the pain and symptoms of FM. Palmitoylethanolamide (PEA) is a well-known endogenous endocannabinoid-like lipid mediator, with neuroprotective, anti-inflammatory and analgesic properties [117]. The endocannabinoid system is an essential endogenous pathway involved in the pathophysiology of chronic widespread pain and FM [118]. A growing amount of the literature supports the potential role of PEA in several health conditions correlated with NcplP. It counteracts mast cells and microglia activation [119,120] via interaction with several targets, including TRPV1 channels [121] and the CB2 pathway [122].

Preclinical investigations demonstrated that PEA, apart from inhibiting degranulation of mast cells in the peripheral tissue, is also able to modulate microglia activation in the CNS [123]. Its activity on glutamate synapses in the mPFC support its role as a potential therapeutic approach for treating chronic pain and the relative negative affective state [124]. Moreover, ultramicronized PEA (um-PEA), which has a diameter of 0.8 ± 2 μm that makes it optimally absorbable along the intestine and able to cross the BBB, was shown to reduce the amount of spinal and hippocampal pro-inflammatory cytokines, with beneficial effects on pain and associated antidepressive and anxiolytic effects [125]. PEA has also been widely studied for its neuroprotective effect in counteracting neuroinflammatory conditions and delaying the progression of neurodegenerative diseases [126].

A retrospective study conducted in patients suffering from FM showed the efficacy and safety of um-PEA, as add-on therapy, in patients treated with duloxetine (DLX) and pregabalin (PGB). After 3 months of treatment, um-PEA introduction provided a significant improvement in pain symptoms and a reduction in the number of tender points compared to combined DLX + PGB only (*p* < 0.0001) [127]. Similarly, a retrospective analysis of 359 FM patients, prescribed with orally um-PEA, showed a statistically significant improvement in pain score (*p* < 0.001) and in FM Impact Questionnaire score (*p* < 0.001), with an optimal tolerability profile. Only 13.7% of patients reported adverse events predominantly of a gastrointestinal type [128].

Other clinical studies evaluated the combination of PEA + L-acetyl carnitine (LAC), which is well-known to exhibit antioxidant and anti-inflammatory properties and to have beneficial effects on different types of neuropathic pain. A randomized control trial evaluated the efficacy of adding PEA + LAC to standard treatment with DLX + PGB in 130 FM patients for a 12-week follow-up. Significant improvement was recorded in all clinical outcomes—the Widespread Pain Index, the patient-completed revised Fibromyalgia Impact Questionnaire and the modified Fibromyalgia Assessment Status questionnaire—in the group treated with the combination PEA + LAC and DLX + PGB [129]. A recent retrospective study supported the hypothesis that the combination PEA + LAC may be mostly effective in a subgroup of FM patients with suspected small fiber involvement [130]. These data confirmed the well-known role of LAC in mitigating peripheral neuropathies, while the central mechanisms of neuroinflammation are mainly controlled by the effects of um-PEA in the CNS. Moreover, the improved answer to treatment in the subgroup with suspected small fiber neuropathy is a clear sign of the urgent need for clustering patients suffering from NcplP in different phenotypes [131,132]. We can speculate that understanding the mechanisms involved in different phenotypes of NcplP may help physicians in selecting a targeted treatment and possibly improve the outcome.

Further clinical investigations are warranted to confirm the role of modulating neuroinflammation in FM management.

Due to its anti-inflammatory, antioxidant, analgesic, and immunomodulatory effects, PEA has shown to have beneficial effects in several gastroenteric diseases. As mast cells implicated in the pathophysiology on irritable bowel syndrome, co-micronized PEA/polydatin (Pol) have been investigated in the treatment of abdominal pain symptoms in patients suffering from IBS. In a randomized clinical trial, PEA/Pol has been shown to significantly improve the abdominal pain intensity in adults with IBS compared with a placebo. Patients suffering from IBS were shown to have a higher mast cell count, a lower expression of reduced fatty acid amide oleoylethanolamide and an increased amount of cannabinoid receptor 2 [133]. A recent multicenter study confirmed these results in 70 children suffering from IBS, where the co-micronized formulation PEA/Pol was shown to be effective and safe, with particular benefit in the IBS-diarrhea subtype [134]. Considering the lack of specific treatments, findings from these studies further support the potential role of um-PEA in modulating neuroinflammation in IBS, which is a well-recognized form of nociplastic pain.

## 7. Discussion

NcplP is a mechanistic term used to describe a type of pain that arises or is sustained by altered nociception, despite the absence of tissue damage [135]. Diagnosis of NcplP has relied for a long time on the use of grading systems, scales and questionnaires intended for other purposes, such as the evaluation of chronic pain [136,137], albeit with low specificity for NcplP itself [138]. In 2022, the IASP endorsed the criteria for NcplP developed by Kosek and colleagues [139], which focused on the presence of features and manifestations that are compatible with central sensitization, such as widespread pain lasting over than 3 months and hypersensitivity to sensory stimuli (namely pressure, movement, heat/cold, touch), alongside comorbidities such as cognitive impairment, fatigue and sleep disturbance, all while ruling out neuropathic and nociceptive pain [140]. Therefore, FM may be considered the prototype of NcplP.

Even though a biomarker is missing, several imaging techniques have been tested for diagnostic purposes in FM, including functional magnetic resonance imaging (MRI) and PET, with the latter exploring different CNS pathways that are potentially associated with FM. In this review, we focused on the role of microglia activation as a marker of neuroinflammation in NcplP, according to data showing the imbalance between pro- and anti-inflammatory microglia in fibromyalgia [141]. Therefore, we analyzed PET imaging studies, where cerebral upregulation of the TSPO can be used for detecting microglia activation, as recently reported in FM subjects. Even though TSPO overexpression is non-specific for FM or NcplP [110], the available evidence from PET neuroimaging supports the hypothesis that such conditions are triggered by neuroinflammatory processes.

Currently, in the field of NcplP, PET imaging techniques are clearly only for research purposes and do not provide any further diagnostic tools for clinical investigation. However, they are useful for understanding the pathophysiology of a “silent disease”, where the delay in diagnosis is a recognized relevant factor in worsening clinical outcomes [25]. However, beside cancer, PET imaging is currently being used, even in other clinical settings, such as in inflammatory and infectious diseases [142,143]. Despite the use of ionizing radiation, the high selectivity and the low concentration (nano-molar to pico-molar range) of required RPs represents a meaningful advantage in clinical practice: in particular, in the relationship with the negligible incidence of media-related adverse events. Therefore, we could also imagine a possible role in the future for the diagnosis of NcplP.

Furthermore, the optimized design of RPs that selectively target TSPO, using biomolecules, would be very useful. To date, most clinically used RPs are labeled peptides or antibodies for various applications. Radiolabeled peptides and peptidomimetics selectively targeting TSPO are currently not available; however, they could be very useful for these applications [144,145,146].

Beyond PET, other imaging modalities, such as MRI, have been investigated in NcplP. MRI does not require ionizing radiation and can be used multiple times for diagnosing and monitoring the effects of treatments. Moreover, significant advantages arise from the reduced costs and worldwide availability of MRI. However, even MRI does not represent the current standard of care for the diagnosis of NcplP.

Understanding the exact mechanism underlying NcplP is a key factor for a targeted therapeutic strategy. Current pharmacological therapies are indeed burdened by several side effects, which may significantly affect the QoL of patients. Moreover, the minor perceived effects of pain relief led to low compliance and a high rate of discontinuation [30]. Different non-pharmacological therapies have been proposed as first line treatments in FM [147], some of which have been shown to have effects on neuroinflammation. Exercise showed promising results against neuroinflammatory processes, both in preclinical models [148,149] and in clinical studies [150]. Psychological interventions, including mindfulness [151] and cognitive behavioral treatment [152], may play an important role in FM management [153] through the modulation of neuroinflammation [154].

When such options are ineffective, pharmacological approaches should be considered [155]. Non-steroidal anti-inflammatory and steroids are not recommended, because they cannot suppress or mitigate neuroinflammatory process drugs [156]. Moreover, their long-term use should be avoided due to the risk of gastrointestinal, cardiovascular and renal side effects. Opioids are part of the pharmacological treatment of chronic pain; however, no efficacy has been demonstrated for NcplP [157]. Traditional strong opioids are discouraged because of warnings about their safety [158]. Nonetheless, dual and multi-mechanistic opioids, such as tramadol, tapentadol or buprenorphine, which also target pain modulatory pathways, could be clinically useful in a small proportion of patients [159,160], since serotonin and norepinephrine have pivotal roles in NcplP [161]. They should be used at the lowest possible dose, for the shortest period of time. Accordingly, antidepressants, such as tricyclic antidepressants and serotonin-norepinephrine reuptake inhibitors, in particular venlafaxine and duloxetine, are currently used as pharmacological options in FM [162]. Among anticonvulsants, PGB and gabapentin [163,164] are the most commonly used and investigated. Both classes of adjuvants counteract neuroinflammatory responses [165,166,167,168].

Recent preclinical and clinical investigations support targeting neuroinflammation, namely activated microglia, as a possible strategy to minimize drug-related adverse effects and to potentiate analgesic effectiveness. Um-PEA results a promising lipid mediator, which may be used as add-on therapy in NcplP, because of its extensively documented anti-inflammatory, analgesic, immunomodulatory, antimicrobial and neuroprotective effects [169].

## 8. Conclusions

NcplP has recently been identified as the new “third mechanism” of chronic pain, but it is still a quite complex entity to be diagnosed, in relation to the lack of specific biomarkers and many different associated pathological diseases. PET neuroimaging has been shown to be a valid technique for understanding the role of neuroinflammation in chronic pain syndromes. Preclinical models and TSPO-targeting RPs supported the hypothesis that microglia activation plays a key role in the pathogenesis of NcplP. Further investigations will clarify the exact role of PET neuroimaging as a clinical tool, for detecting and managing therapeutic strategies in NcplP conditions.

## Figures and Tables

**Table 1:** Summary of available studies investigating TSPO-targeting RPs for PET imaging in FM patients, for detecting microglia activation as a marker of neuroinflammation.

TSPO-Targeting RPs	Population	Main Findings	Ref.
[^11^C]PBR28	31 FM (29 females) 27 HCs (25 females)	FM patients exhibited the following: Widespread cortical elevations in TSPO binding (mainly in the medial and lateral walls of the frontal and parietal lobes).	[100]
[^11^C]PK11195	12 FM (5 females) 11 CRPS (3 females) 15 HCs (5 females)	FM patients exhibited the following: - Increased neuroinflammation in the precentral and postcentral gyrus; - Lower neuroinflammation in the medulla, left amygdala and left superior temporal gyrus.	[101]
[^18^F]DPA-714	31 FM 18 HCs	FM patients exhibited the following: - Increased bilateral neuroinflammation in the precuneus, postcentral gyri, parietal and occipital GM, supramarginal gyri; - Increased neuroinflammation in the right temporal GM and the left isthmus of the cingulate gyrus.	[102]

## Data Availability

No new data were created or analyzed in this study. Data sharing is not applicable to this article.

## Abbreviations

The following abbreviations are used in this manuscript:

ACC	Anterior Cingulate Cortex
AD	Alzheimer’s Disease
BBB	Blood–Brain Barrier
BDNF	Brain-Derived Neurotrophic Factor
CatS	Cathepsin S
CGRP	Calcitonin Gene-Related Peptide
CNS	Central Nervous System
CRPS	Complex Regional Pain Syndrome
CSF	Cerebrospinal Fluid
DLX	Duloxetine
FM	Fibromyalgia
GM	Gray Matter
HCs	Healthy Controls
IASP	International Association for the Study of Pain
IBS	Irritable Bowel Syndrome
IC	Interstitial Cystitis
IgG	Immunoglobulin G
IL	Interleukin
LAC	L-Acetyl Carnitine
mPFC	Medial Rostral Prefrontal Cortex
MRI	Magnetic Resonance Imaging
MS	Multiple Sclerosis
NcplP	Nociplastic Pain
NGF	Nerve Growth Factor
PD	Parkinson’s Disease
PEA	Palmitoylethanolamide
PET	Positron Emission Tomography
PGB	Pregabalin
Pol	Polydatin
QoL	Quality of Life
RCS	Repeated Cold Stress
RPs	Radiopharmaceuticals
TNF	Tumor Necrosis Factor
TSPO	Translocator Protein
